# The Discovery of Naringenin as Endolysosomal Two-Pore Channel Inhibitor and Its Emerging Role in SARS-CoV-2 Infection

**DOI:** 10.3390/cells10051130

**Published:** 2021-05-07

**Authors:** Antonella D’Amore, Antonella Gradogna, Fioretta Palombi, Velia Minicozzi, Matteo Ceccarelli, Armando Carpaneto, Antonio Filippini

**Affiliations:** 1 Unit of Histology and Medical Embryology, Department of Anatomy, Histology, Forensic Medicine and Orthopaedics, Sapienza University of Rome, 16 Via A. Scarpa, 00161 Rome, Italy; antonella.damore@uniroma1.it (A.D.); fioretta.palombi@uniroma1.it (F.P.); 2 Institute of Biophysics, National Research Council, Via De Marini 6, 16149 Genova, Italy; 3 INFN and Department of Physics, University of Rome Tor Vergata, Via della Ricerca Scientifica 1, 00133 Roma, Italy; velia.minicozzi@roma2.infn.it; 4 Department of Physics, University of Cagliari, 09042 Monserrato, Italy; matteo.ceccarelli@dsf.unica.it; 5 IOM-CNR Unità di Cagliari, Cittadella Universitaria, 09042 Monserrato, Italy; 6 Department of Earth, Environment and Life Sciences (DISTAV), University of Genoa, Viale Benedetto XV 5, 16132 Genova, Italy

**Keywords:** TPC channels, plant vacuole, mammalian endolysosome, flavonoids, coronavirus, SARS-CoV-2

## Abstract

The flavonoid naringenin (Nar), present in citrus fruits and tomatoes, has been identified as a blocker of an emerging class of human intracellular channels, namely the two-pore channel (TPC) family, whose role has been established in several diseases. Indeed, Nar was shown to be effective against neoangiogenesis, a process essential for solid tumor progression, by specifically impairing TPC activity. The goal of the present review is to illustrate the rationale that links TPC channels to the mechanism of coronavirus infection, and how their inhibition by Nar could be an efficient pharmacological strategy to fight the current pandemic plague COVID-19.

## 1. Introduction

TPC channels (TPCs) are intracellular membrane channels found in both plant and animal cells, from echinoderms to humans, with different isoforms, the phylogenetic history of which is partially characterized [1,2,3,4,5,6] In plant cells, they are located on the membrane of the vacuole, the so-called tonoplast [1,7]. The vacuole is a peculiar compartment of plant cells that, in mature cells, can occupy more than 80% of the volume [8]. The physiological role of plant TPCs has not yet been determined; different hypotheses have been proposed, including their involvement in a calcium-induced calcium released mechanism [9], since they are cationic channels with a significant calcium permeability [10,11,12], activated by an increase of cytosolic calcium concentration. They could also be involved in the maintenance of potassium homeostasis and in the compartmentalization of sodium ions [13]. Recently, it has been shown that they are able to confer electrical excitability to the tonoplast [14] similar to that elicited by human TPC1 in endolysosomal membranes [15]; however, it is not clear how this excitability can be translated into a signal of physiological relevance. Other experiments indicate that they are involved in signal transduction chains leading plants to respond to abiotic and biotic stresses [16,17,18,19].

Human TPC channels (TPC1 and TPC2) are located on the membrane of the endolysosomal compartments, are important in trafficking mechanisms and homeostasis of endolysosomes [2,3,4], have been related to a number of pathologies such as Parkinson’s disease, nonalcoholic fatty liver disease, virus infection, cancer, diabetes, and cardiac dysfunction [20,21], and have emerged as important players in neoangiogenesis [22,23]. In the present work, we will show the steps that, starting from plant TPC channel functional characterization, led us to formulate and validate (in vitro) the hypothesis that human TPCs are involved in the mechanism of viral infection mediated by coronaviruses and that their inhibition by Nar has the potential to be a powerful anti-SARS-CoV-2 pharmacological weapon.

## 2. Plants and TPC Channels

**Figure 1:**
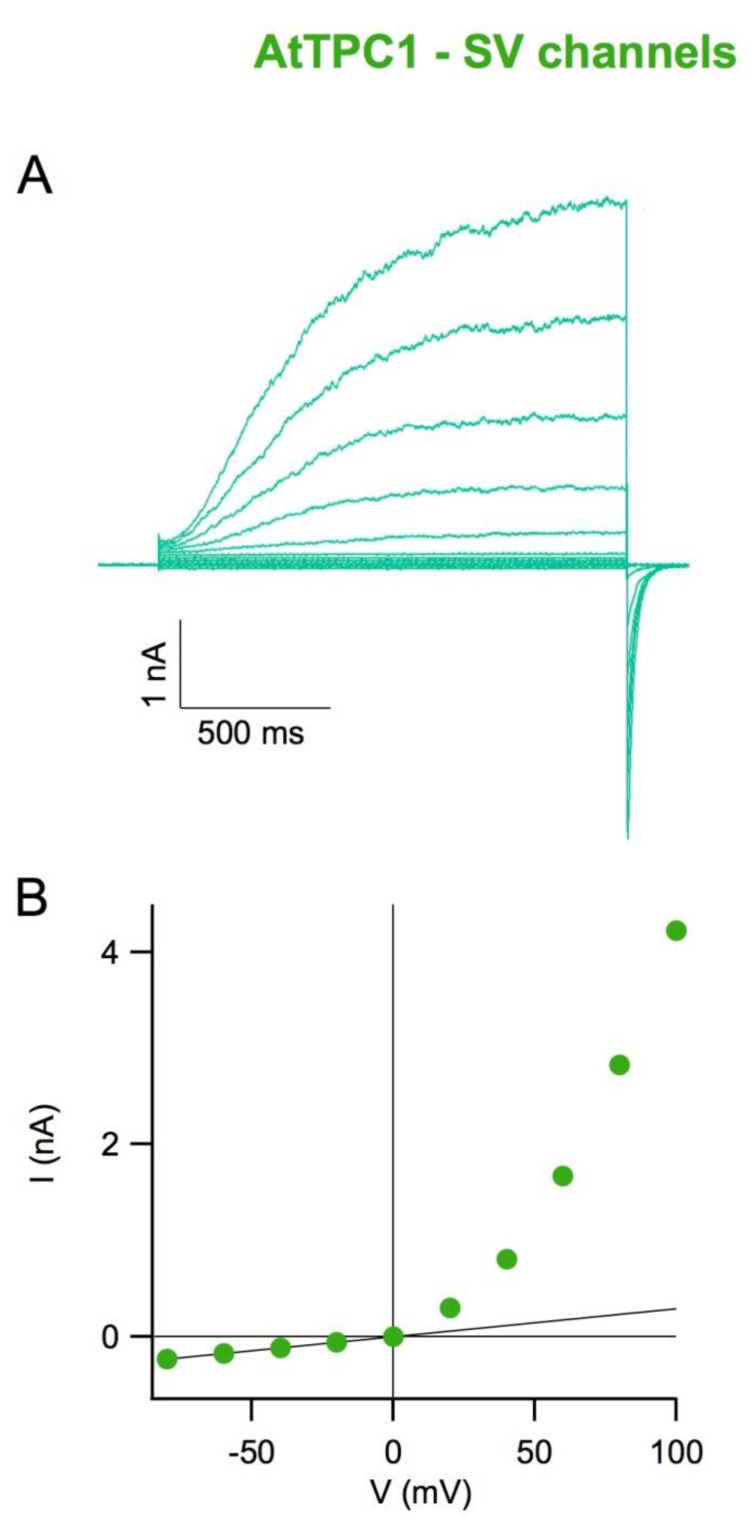
Slow Vacuolar channels in plant vacuoles. (**A**) AtTPC1–SV currents recorded in a vacuole isolated from Arabidopsis mesophyll cells in excised cytosolic side-out patch configuration. Currents were elicited by a series of voltage steps ranging from −80 mV to +100, in 20 mV steps. Holding potential −50 mV, tail potential −50 mV. Pipette solution (in mM); 200 KCl, 2 MgCl_2_, 2 CaCl_2_, 10 MES/Tris, pH 5.5; bath solution (in mM): 100 KCl, 2 MgCl_2_, 1 CaCl_2_, 1 mM dithiothreitol (DTT), and 10 mM HEPES/Tris, pH 7.5. The osmolarity in both solutions was adjusted to 600 mOsm by the addition of D-sorbitol. (**B**) SV current–voltage characteristics of the traces shown in panel A. The I–V characteristics are constructed by plotting the average value of the currents recorded during the last 50 ms at each applied voltage. Positive currents represent cations entering the vacuole. The continuous black line represents the background current evaluated from the linear fitting of the current between −80 and 0 mV.

The first detailed functional characterization of ion channels belonging to the TPC family was performed in 1987 by Rainer Hedrich during his PhD studies in the laboratory of the Nobel Prize for Medicine Erwin Neher [24]. The experimental preparation consisted of vacuoles isolated from sugar beet roots; a homogeneous portion of sugar beetroot was cut with a scalpel and then washed with an ionic solution with osmolarity equal to that measured in the root. The patch-clamp technique in the whole-vacuole configuration was applied to the vacuoles thus obtained. By applying positive membrane potentials, positive currents similar to those shown in Figure 1A were recorded. It can be observed that currents have very slow activation and deactivation times; for this reason, these channels have been given the name slow vacuolar (SV) channels. I–V characteristics of Figure 1B indicate that these are outward rectifier channels. Selectivity experiments have shown that these channels are cationic with a similar permeability for potassium (generally the physiologically relevant ions in plant cells) and sodium ions [24,25]. These channels also have significant permeability for calcium divalent ions [10,11]. A very interesting property of the channels is that in the absence of cytosolic calcium (Ca^2+^ < 1 µM), they turn out to be closed [24]; an increase in cytosolic calcium concentration leads to an increase in channel activity [24]. Single-channel conductance is high, with values of about 100 pS in symmetrical potassium concentrations, equal to 150 mM [26].

Several studies have shown that these channels are present in all types of plant cells and in all plants investigated so far, even in marine [25,27] and freshwater [28] plants. They also undergo a variety of modulations including dependence on the stimulation protocol [29], cytosolic magnesium [30], heavy metals [28,29,31,32], the antibiotic neomycin [33], polyamines [34,35], ruthenium red [36], and PUFAs [37].

## 3. The SV Channels Are Modulated by Redox Agents and Flavonoids

At physiological potentials of the plant vacuole, around −30 mV [1], the SV channel is essentially closed even at cytosolic calcium concentrations of 1 mM. Therefore, it has been hypothesized the presence of a “helper factor” capable of shifting the voltage dependence of the channel to more negative potentials [38]. Experiments conducted on vacuoles isolated from the marine plant *Posidonia oceanica* clearly indicated that the SV channel needed a reducing environment on the cytosolic side to be active [27]. The reducing agent DTT or endogenous antioxidants such as glutathione and ascorbate were necessary for the functioning of the SV channel [27], while the presence in the cytosolic solution of oxidants such as chloramine-T or the SH-group modifying agent phenylarsine oxide (PAO) led to its irreversible inhibition [39,40,41].

Since flavonoids, a very large class of plant secondary metabolites, are known to have antioxidant properties [42], we checked whether naringenin could work as a helper factor for the SV channel. However, in vacuoles isolated from carrot roots naringenin concentrations above 100 µM behaved similarly to a reversible channel inhibitor [43]. 

## 4. The SV Channel Protein in *Arabidopsis thaliana* Is Encoded by the *TPC1* Gene 

In 2005, it was discovered that, in the *Arabidopsis thaliana* model plant, the TPC1 gene, the only gene of the TPC channel family in *Arabidopsis*, encodes the protein that mediates the SV-type currents [44]. In TPC1 knockout vacuoles, the SV currents were totally absent. However, KO plants do not exhibit any phenotype, compared to WT plants. 

**Figure 2:**
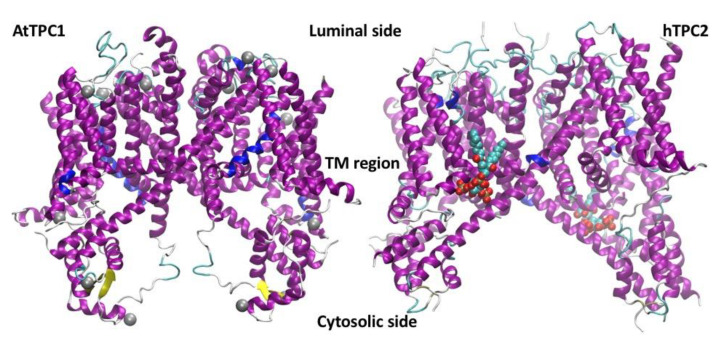
Molecular structures of AtTPC1 and hTPC2. (LEFT) Side view of the high-resolution tridimensional structure of AtTPC1 (X-ray at 2.87 Å, pdbid: 5dqq) with the bound calcium ions as gray spheres. (RIGHT) Side view of the high-resolution tridimensional structure of hTPC2 (cryo-EM at 3.7 Å, pdbid:6dq0) with the PI(3,5)P_2_ effector bound to the two homodimers represented as van der Waals spheres. The two proteins differ for the presence of the EF hands in AtTPC1 on the cytosolic side (bottom in the figure), able to bound calcium ions.

AtTPC1 is a 733 amino acid protein formed by two shaker-type units joined by a cytosolic linker that has two EF-hands domains capable of binding cytosolic calcium [45]. Each shaker-type domain consists of six transmembrane segments: between the fifth and sixth segments, there is a loop, called P, responsible for the formation of the permeation pore. Since four P-loops form a functioning pore, the TPC1 channel assembles as a homodimer. The S4 segments of the individual shaker units possess basic amino acids capable of functioning as sensors of the membrane potential. Structural data obtained from TPC1 crystals [46,47,48,49] indicate that only the S4 segment belonging to the second shaker unit forming the monomer contributes to the voltage-dependent gating, unlike the typical voltage-dependent potassium, sodium, or calcium channels in which all S4 segments contribute to the transduction of the membrane potential in channel opening. A comparison of the molecular structures of AtTPC1 and hTPC2 [50] is shown in Figure 2. Interestingly, AtTPC1 is also inhibited by the presence of the flavonoid naringenin in the cytosolic solution [23].

## 5. Plant Vacuoles as a Heterologous System of Expression and Characterization of Human TPCs 

**Figure 3:**
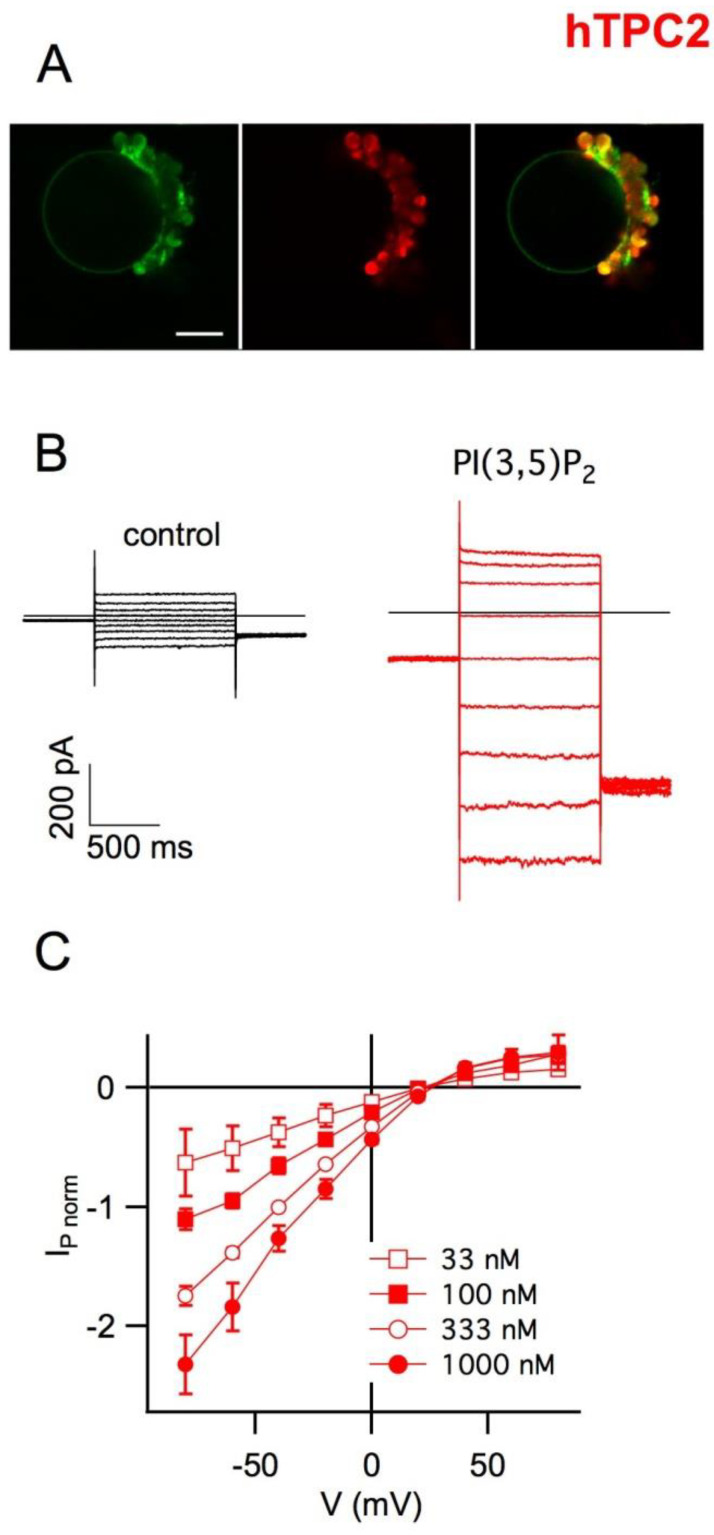
Functional characterization of human TPC2in plant vacuole as heterologous system (**A**) Vacuolar membrane localization of hTPC2-EGFP in mesophyll protoplasts from *Arabidopsis* lacking endogenous *tpc1*. Confocal fluorescence images of an isolated vacuole expressing hTPC2-EGFP on the tonoplast. Left: EGFP signal (green); middle: chlorophyll signal (red); right: merge. Scale bar: 7 µm. (**B**) Whole-vacuolar current recordings in control conditions (black traces; left) and in the presence of 100 nM PI(3,5)P_2_ in the bath solution (red traces; right), elicited by 1-s voltage pulses from +80 to −80 mV in 20 mV decrements. (**C**) Current–voltage relationships of PI(3,5)P_2_-evoked hTPC2 currents (I_P_) as shown in (**B**). For each vacuole, current amplitudes determined at different [PI(3,5)P_2_] were normalized to the value at −40 mV in the presence of 330 nM PI(3,5)P_2_. Figure modified from [59] with kind permission from Springer Nature Customer Service Center GmbH (license number5046950331855).

**Figure 4:**
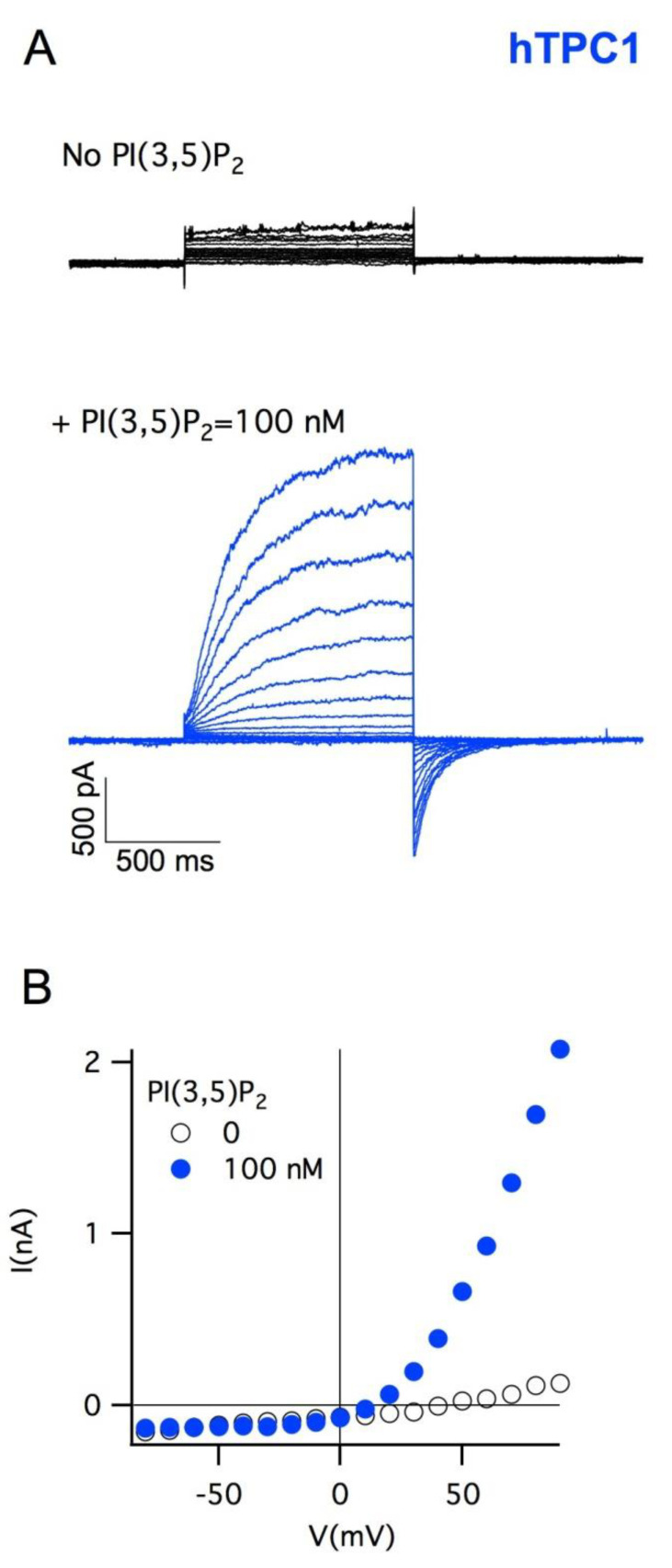
Human TPC1 is functional when transiently expressed in vacuoles from mesophyll cells of *Arabidopsis* plants lacking endogenous *tpc*. (**A**). Currents recorded at different voltages, from −80 mV to 90 mV, step +10 mV, in a symmetrical concentration of sodium (100 mM), respectively, in the absence and in the presence of 100 nMPI(3,5)P_2_ show that hTPC1 is activated by this phosphoinositide. Tail pulse at −50 mV. (**B**) From the I–V characteristics of the currents displayed in A, it is evident that hTPC1 is a voltage-dependent, outward-rectifying channel. The standard pipette (luminal side) solution contained (in mM): 100 NaCl, 2 MgCl_2_, 1 CaCl_2_, 10 MES, pH 5.5 (with NaOH). The standard bath (cytoplasmic side) solution contained (in mM): 100 NaCl, 10 Hepes, pH 7.5 (with NaOH). The osmolarity of the luminal and cytoplasmic solutions was adjusted to 550 mOsm and 600 mOsm, respectively, by the addition of D-sorbitol.

Plant vacuole plays a fundamental role in cellular homeostasis, among the various physiological functions, it can be considered the warehouse in which the cell preserves its metabolites [51]. From a technical point of view, the plant vacuole is simple to isolate, and its dimensions can reach up to 40 µm in diameter: these characteristics make it ideal for applying the patch-clamp technique. It can be used as an alternative to planar membranes to characterize the functional activity of channel-forming peptides (CFP) [52,53,54,55]. We verified that endolysosomal animal transporters could be successfully expressed in the vacuolar membrane [56]. In order to obtain this result, we isolated by enzymatic treatment protoplasts from the leaf mesophyll of *Arabidopsis*; we used a well-defined transient protoplast transfection protocol [57] with a plasmid [58] containing the sequence of the CLC-7 endolysosomal rat transporter fused to its C-terminus to a GFP [56].To avoid interferences with endogenous proteins, we used *Arabidopsis* KO plants for AtCLCa, the plant homolog of CLC-7. After about 40 h from transfection, we verified by detection of the fluorescence emitted by GFP that the transporter had reached the tonoplast; patch-clamp experiments showed that CLC-7 was working and operating as an antiport that exchanged a proton for 2 chloride ions [56]. The same approach was followed to express the two human channels TPC2 [59] and TPC1 [60], again in vacuoles isolated from the leaf mesophyll of Arabidopsis mutants, this time lacking the endogenous channel AtTPC1. In Figure 3, it can be observed that human TPC2 fused to EGFP was targeted to the membrane of the large central vacuole and that hTPC2-mediated currents were activated by nanomolar concentrations of the phosphoinositide PI(3,5)P_2_ [61] and did not have a strong voltage dependence. The plant homolog AtTPC1 is not modulated by PI(3,5)P_2_ [61], which acts as a high-affinity inhibitor (nanomolar range) of tonoplast anion (AtCLCa [62]) and cation (AtNHXs [63]) transporters. Human TPC1 is also functional when expressed in vacuoles (Figure 4); similarly to hTPC2, nanomolar concentrations of PI(3,5)P_2_ are required to activate hTPC1. As revealed by molecular simulations, the phosphoinositide binding activates anticorrelated movements of the two units, allowing the opening of the gate region, constituted by two rings of hydrophobic residues on the cytosolic side [64]. It is worth noting how this region represents the bottleneck for the diffusion of ions that move partially hydrated. Moreover, hTPC1 turns out to be an outward-rectifier, voltage-dependent channel, as shown in the I–V characteristics of Figure 4B.

## 6. The Effect of the Flavonoid Naringenin on Neoangiogenesis

As mentioned in the previous section, the plant vacuole can be an efficient heterologous system for functional characterization of intracellular TPC channels, similar to *Xenopus* oocytes [65,66,67,68] or cell cultures [69] for plasma membrane ion channels and transporters. We wondered if the flavonoid naringenin could modulate TPC channel-mediated currents. We found that naringenin is capable of inhibiting both hTPC2 and hTPC1 at concentrations of hundreds of micromolar [23]. The presence of hTPC2 has been shown to be essential for neoangiogenesis [22]; this phenomenon of generation of novel vessel-like structures is used to support tumor growth and tumor cell survival. We demonstrated the ability of naringenin to inhibit the formation of vessel-like structures upon VEGF stimulation in vitro and in vivo [23]. Moreover, it has been demonstrated that Nar can inhibit the formation of subintestinal vessels (SIVs) in vivo in zebrafish, indicating its potential antiangiogenic effect [70].

The next step will be to verify whether naringenin is able to inhibit this important process for the development of solid tumors and reduce a common effect associated with an aggressive tumor phenotype, vasculogenic mimicry. In this phenomenon, the tumor cells mimic endothelial cells in the formation of vessel-like structures to help the tumor growth [71]. Founding a novel strategy to inhibit this novel potential therapeutic target in malignant tumors remains necessary for cancer research. 

## 7. Inhibition of TPCs by Naringenin as an Option to Fight Viral Infections

It has been demonstrated that naringenin can impair different viral infections. Dengue is a mosquito-borne viral disease widespread in tropical and subtropical regions throughout the world. Frabasile et al. demonstrated that naringenin can inhibit the infection and the replication and/or maturation of four different Dengue virus (DENV) serotypes in hepatocarcinoma cells Huh 7.5 and impair the infection of DENV-4/TVP360 subtype in the peripheral blood mononuclear cells (PBMCs) [72]. Moreover, Nar can impair Zika virus infection in human lung adenocarcinoma epithelial A549 cells [73]. Nar activity is a lineage-independent activity for this kind of virus. Indeed, there are two different lineages of the Zika virus, the African and the Asian, but Nar is effective on both of them. Furthermore, molecular docking has been used to explain the mechanism of action of this flavonoid. Nar may act as a noncompetitive inhibitor of the NS2B-NS3 Zika viral protease [73]. Hepatitis C virus (HCV) infection is the main cause of chronic liver disease around the world. It has been demonstrated that Nar inhibits HCV production, blocking the assembly of viral particles [74]. Chikungunya virus (CHIKV) is a mosquito-transmitted alphavirus. Two different research groups [75,76] demonstrated the effect of Nar on CHIKV infection. In particular, it has been demonstrated that Nar inhibited postentry stages of CHIKV, impairing the accumulation of nonstructural proteins (nsP1-nsP3) that are virus-specific RNA replicase subunits [76]. Regarding TPCs, their role in Ebola infection has been demonstrated; knockdown or knockout of either TPC1 or TPC2 can block Ebola infection in vitro. Moreover, another plant-derived TPCs inhibitor, tetrandrine, was used in this study proving the efficacy in vivo in a mouse model of Ebola infection [77]. This background emphasizes the importance of a natural compound such as Nar against viral infection. Almost 10 clinical trials are registered at clinicaltrial.gov regarding Nar, and its safety has been reviewed [78]. Moreover, a recent pharmacokinetic and metabolic study reported its safe use in clinical studies [79]. Of note, in healthy humans, a serum concentration of 50 µM did not show relevant toxicity, considering its equivalence to an oral dose of 600 mg of Nar [80].

## 8. Naringenin Is a Powerful Anti-Coronavirus Drug In Vitro

Coronaviruses (CoV) are enveloped viruses containing positive-strand RNA, the genome is complexed with the basic nucleocapsids (N) protein to form a helical capsid within the membrane. To the membrane are associated almost three different proteins such as spike (S), a type I glycoprotein giving the virus its crown-like morphology, the membrane (M) protein, and the E protein, strongly hydrophobic. In this family, we can distinguish the highly pathogenic SARS-CoV-1 and -2, MERS-CoV, and other four human CoV (229E, OC43, HKU1, NL63), which cause usually respiratory illness in humans [81]. CoV infection is correlated to virions trafficking to lysosomal compartments where the lysosomal protease processes S protein allowing the virus entry. 

We were the first research group that proposed the TPC channels as molecular targets to inhibit SARS-CoV-2 infection [82]. Later, this hypothesis was also formulated by others [4,83,84], and experiments carried out by Ou et al. [85] showed that tetrandrine, an inhibitor of TPCs, significantly decreased the entry of SARS-CoV2 pseudovirion on HEK 293 cells, expressing the human angiotensin-converting enzyme 2 (ACE2), the main cell entry receptor used by SARS-CoVs. 

We performed experiments in the human cell line Huh7.5 pretreated with siRNA to silence TPC2 [86]. TPC2 silenced and control cells were then infected with the coronavirus HCoV 229E: infection in TPC2 knockdown cells was significantly inhibited, compared to control, strongly indicating an active role of TPC2 in the mechanisms of coronavirus infection. In line with these results, we could verify a strong antiviral activity of naringenin, which was able to inhibit, in vitro, the infection by three different human coronaviruses, HCoVOC43, HCoV229E, and, very interestingly, SARS-CoV-2 [86]. The high concentration of naringenin (hundreds of micromolar) effective in contrasting coronavirus infection matched the Nar affinity constant of TPC2 inhibition [23], suggesting that TPC2 could be the molecular target of Nar. 

Interestingly, a very recent article showed that Nar, as well as two other flavonoids, specifically inhibited hTPC2 but not the endolysosomal cation channel TPRML1 [87]. Despite the vaccines, finding a drug to fight Coronavirus disease 19 (COVID-19) remains an important goal of our research. Nar could be an interesting tool, given its role in the regulation of immune responses (reviewed in [88] and referenced in Table 1) and in decreasing ACE2 expression in rat kidneys [89]. Nar can regulate cytokine release from macrophages and T cells such as TNF alpha and IL-6. In particular, this phenomenon is lysosome dependent since bafilomycin and NH_4_Cl treatment, which raise lysosomal pH, blunt Nar effect [90]. In addition, it has been demonstrated that Nar can influence CD4^+^ T cell proliferation and can inhibit helper T cell (Th) 1 and Th17 differentiation: both these cells are proinflammatory subsets that promote the development of autoimmunity and tissue damage [91]. Indeed, SARS-CoV-2 could act as a triggering factor for the development of a rapid autoimmune and/or autoinflammatory dysregulation, leading to severe interstitial pneumonia [92]. Moreover, a common feature in the COVID-19 severe patients is related to an exacerbation of neutrophil activation [93]. Nar can reduce neutrophils infiltration reducing airway inflammation and lung injury, in a mouse model of acute respiratory distress syndrome (ARDS) [94].

All these data allow us to single out Nar as a pharmacological blockade of SARS-CoV-2 infectivity, as claimed by us since the beginning of the first pandemic lockdown in Italy [82].

## 9. Perspectives and Conclusions

**Figure 5:**
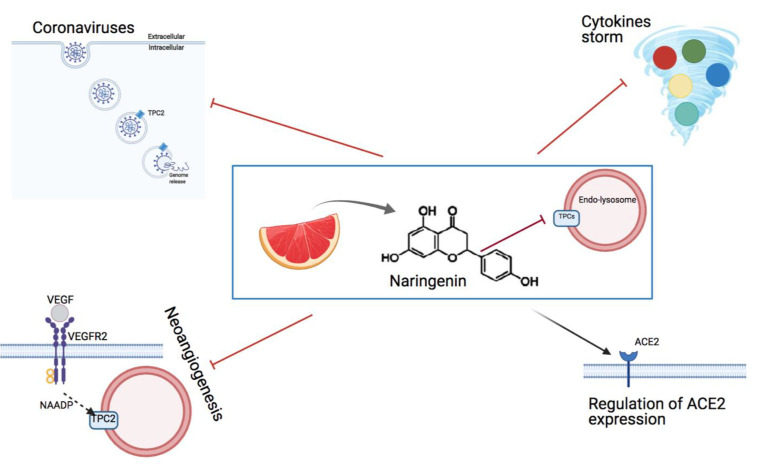
Schematic representation of the possible targets of the flavonoid naringenin, an effective inhibitor of TPCs.

TPCs are involved in different diseases (virus infection, Parkinson’s disease, cancer, diabetes, cardiac hypertrophy) and are becoming an important key point for the individuation of a novel therapeutic target. Thus naringenin, an effective inhibitor of TPCs, could be an up-to-date approach according to the several clinical trials performed and still ongoing (Figure 5). A further step will require the molecular characterization of the specific binding site to better understand the mechanism of action of this molecule. This will be useful to develop effective drugs to inhibit TPCs activity when required. 

## Figures and Tables

**Table 1:** Notable regulatory effects of naringenin on the immune response.

Naringenin (Concentration/Dose) and Model	Immune Regulation Effects	References
50 mg/kg Hepatocytes/Hypercholesterolemia	Naringenin reduces TNF- α, IL-6, and IL-1β by suppressing NF-kB.	[95]
100 µM Pre-polarized M1 macrophages	Naringenin reduces TNF- α production.	[96]
50–100 µM T cells 100 µM Macrophages	Naringenin reduces TNF- α, IL-6 secretion regulating cytokines degradation through lysosome-TFEB dependent mechanisms.	[90]
100 mg/Kg Mouse model of ARDS	Naringenin reduces neutrophil infiltration reducing airway inflammation and lung injury.	[94]
100 mg/Kg in vivo and in vitro studies	Naringenin reduces Monocyte chemoattractant protein (MCP)1 secretion suppressing macrophages infiltration in adipose tissue.	[97]
25–50 µg/mL Macrophages and ex vivo whole blood	Naringenin reduces proinflammatory cytokines (IL-8, IL-6, IL-1β, TNF-α) in macrophages and ex vivo whole blood samples.	[98]
80 µM CD4^+^T cells	Naringenin inhibits Th1 and Th17 differentiation.	[91]
